# Alport Syndrome Presenting as Incidental Finding of Proteinuria on Pre-Employment Checkup: A Case Report

**DOI:** 10.1155/crin/9933123

**Published:** 2025-02-28

**Authors:** Abigayle Therese R. Guiritan, Lee-Boyd D. Valencia, Sonia L. Chicano

**Affiliations:** St. Luke's Medical Center, Quezon City, Philippines

**Keywords:** Alport syndrome, glomerulonephritis, proteinuria

## Abstract

**Introduction:** Glomerulonephritis is a prominent cause of chronic kidney disease and encompasses a subset of renal diseases characterized by immune-mediated damage to the basement membrane, the mesangium, or the capillary endothelium. Symptoms in early stages are usually nonspecific and can be easily overlooked. Unfortunately, if not detected early, this may lead to end-stage renal disease. We present a case of a 23-year-old male patient with no family of kidney disease who had proteinuria on routine urinalysis.

**Case Presentation:** A 23-year-old male, nonhypertensive and nondiabetic, with no family history of kidney disease coming in for proteinuria. During pre-employment checkup, patient was noted to have 4+ proteinuria on urinalysis. Creatinine was requested by company doctor with result of 1.05 mg/dL (eGFR: 104 mL/min/1.73 m^2^). Repeat urinalysis was done but still with 4+ proteinuria on urinalysis. Hence, advised consult with a nephrologist due to persistence of proteinuria. Upon consult, workups were done, which revealed hyperuricemia, urate crystals on urinalysis, persistence of 4+ proteinuria, and urine protein creatinine ratio of 2.8 (urine protein: 223.28 mg/dL and urine creatinine: 79.64 mg/dL). Patient was started on ACE inhibitor, hypouricemic agent, and advised kidney biopsy for further evaluation of proteinuria. The review of systems was pertinent for hearing impairment and blurring of vision. Kidney biopsy was done in which electron microscopy showed segmental podocyte foot process effacement. The glomerular basement membrane shows lamellation and alternate thickening and thinning. No definite electron-dense deposits are seen in glomerular basement membrane and mesangium. Mean glomerular basement membrane thickness is 299 nm (normal mean glomerular basement membrane thickness in adult males is 373 ± 42 nm). He was advised consult with an ophthalmologist and otolaryngologist. Regular checkup, monitoring of renal parameters, and appropriate medications were given.

**Conclusion:** Although a rare cause of glomerulonephritis, Alport syndrome must be considered in patients presenting with subnephrotic range proteinuria and microscopic hematuria. Thorough history and physical examination and characteristic findings on kidney biopsy can help in the prompt diagnosis of the disease. Multidisciplinary care and early intervention can improve the quality of life and delay the progression to end-stage kidney disease among these patients.

## 1. Introduction

Glomerulonephritis is a prominent cause of chronic kidney disease and encompasses a subset of renal diseases characterized by immune-mediated damage to the basement membrane, the mesangium, or the capillary endothelium, resulting in hematuria, proteinuria, and azotemia [1]. Incidence of primary glomerulonephritis vary between 0.2/100 000/year and 2.5/100 000/year [2]. Alport syndrome is characterized by progressive glomerulonephritis, progressive high-tone hearing loss, and visual impairment [3]. It is a rare hereditary disease accounting for 0.2% of adults with end-stage renal disease (ESRD) in the United States [4]. In China, a histopathological study on the spectrum of chronic kidney diseases for the hereditary and congenital diseases showed majority of Alport syndrome (49.6%) [5]. While in Southeast Asia, a seemingly low prevalence of Alport syndrome may be attributed to lack of genetic testing. In fact, recent genetic studies on proteinuric diseases revealed that a third of patients with pathogenic variants for Alport syndrome did not present with features suggestive of Alport syndrome [6]. In the Philippines, although some case reports have been published, data on the prevalence of Alport syndrome are lacking. Symptoms in early stages are usually nonspecific and can be easily overlooked. Unfortunately, if not detected early, this may rapidly progress to ESRD. We present a case of a 23-year-old male patient with no family of kidney disease who had proteinuria on urinalysis.

## 2. Clinical Presentation

This is a case of a 23-year-old male nonhypertensive, nondiabetic, coming in for proteinuria. Three years prior, the patient was apparently well except for frothy urine. He has no frequency, no dysuria, no edema, no decrease in urine output, and no gross hematuria. He consulted a general practitioner and was managed as urinary tract infection. In the interim, there was still persistence of frothy urine with no other accompanying symptoms but no further workup was done. During his pre-employment checkup, there was 4+ proteinuria on urinalysis. Creatinine was requested by the company doctor with a result of 1.05 mg/dL (eGFR 104 mL/min/1.73 m^2^). Repeat urinalysis done but still with 4+ proteinuria on urinalysis. Hence, advised consult with a nephrologist due to persistence of frothy urine and proteinuria. Upon consultation with a nephrologist, workups were done which revealed hyperuricemia, urate crystals on urinalysis, persistence of 4+ proteinuria, and urine protein creatinine ratio of 2.8 mg/dL. Patient was started on ketoanalogues, enalapril, atorvastatin, fish oil, and febuxostat. Further workup revealed elevated ASO titer. Initial impression was glomerulonephritis to consider poststreptococcal glomerulonephritis rule out IgA nephropathy. Amoxicillin clavulanic acid antibiotic was started and 2D echo with doppler was done, which showed unremarkable results. He was then advised kidney biopsy for further evaluation of proteinuria.

There was no recent history of upper respiratory tract infection or skin disease. He is not known hypertensive or diabetic. He has no history of tuberculosis, autoimmune disease, malignancy, or any hepatitis infections. He was previously diagnosed with anxiety disorder and was prescribed with escitalopram as needed. He has visual impairment but no consult done with an ophthalmologist. He was prescribed with reading glasses from an optometrist. He has no family of any kidney or autoimmune diseases.

**Figure 1:**
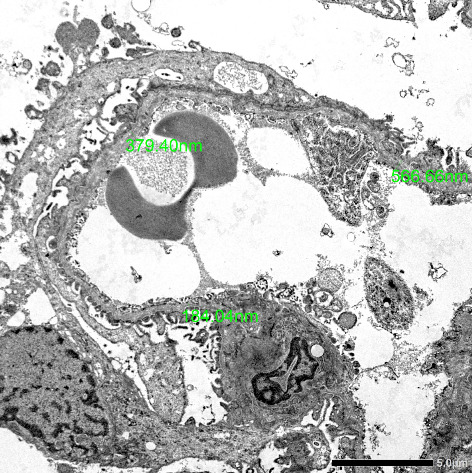
Electron microscopy. Electron microscopy photography showing lamellation or multilayering of the glomerular basement membrane.

Upon consult, the patient was seen awake, not in acute cardiorespiratory distress with stable vital signs and normal anthropometrics. The patient had no bipedal edema and, otherwise, unremarkable physical examination. Kidney biopsy was done in which electron microscopy showed segmental podocyte foot process effacement. The glomerular basement membrane shows lamellation and alternate thickening and thinning. No definite electron-dense deposits are seen in glomerular basement membrane and mesangium. Mean glomerular basement membrane thickness is 299 nm (normal mean glomerular basement membrane thickness in adult males is 373 ± 42 nm) (Figure 1). This finding was suggestive of a glomerular basement membrane abnormality suspicious for Alport syndrome. Final diagnosis was chronic kidney disease G1 A3 secondary to glomerulonephritis secondary to Alport syndrome. He was advised to seek consult with an ophthalmologist and otolaryngologist. He consulted an ophthalmologist and was diagnosed with error of refraction. Visual acuity was 10/200 on the right eye and 20/100 on the left eye. Pure tone audiometry was also done, which suggested bilateral moderate to moderately severe sensorineural hearing loss. Regular checkup and monitoring of renal parameters were also done. He was maintained on enalapril, febuxostat, dapagliflozin, spironolactone, and atorvastatin.

## 3. Discussion

Alport syndrome is a rare genetic disorder resulting from the mutations in the genes COL4A3, COL4A4, and COL4A5 that affect the synthesis, assembly, deposition, or function of the glomerular basement membrane [7]. Although the hallmark of the disease is the presence of hematuria early in life, its presentation can manifest differently among individuals. Early diagnosis and prompt intervention can delay the progression of kidney disease and improve the quality of life in patients with Alport syndrome. Although the interventions currently available are not curative, they can dramatically delay progression to kidney failure, especially if initiated before there is any reduction in glomerular filtration rate (GFR) [8]. In a study done by Watson et al., the use of angiotensin receptor blocker (ARB) can delay the progression of chronic kidney disease or ESRD by reducing intraglomerular pressure and proteinuria, and despite normal renal functions, initiating therapy with ARBs has been shown to have a significant impact on the development of ESRD [4]. In a preclinical study done by Zhu et al., experiments in mice suggested that triple blockade of renin-angiotensin system (RAS), sodium glucose transporter 2 (SGLT2), and mineralocorticoid receptor (MR) may substantially improve renal outcomes in Alport syndrome and possibly other progressive chronic kidney diseases because of synergistic effects on the glomerular and tubulointerstitial compartments [9]. A pilot study among patients with Alport syndrome was also done regarding the use of mineralocorticoid receptor antagonist in addition to RAS and SGLT2 inhibition, which showed an appreciable reduction in proteinuria when used concurrently [10]. In patients who progressively developed to ESRD, long-term hemodialysis or kidney transplantation can be offered. For patients who undergo kidney transplantation, it has been shown that patients with ESRD have similar patient and graft survival to those with other causes of ESRD [11].

## 4. Conclusion

Although a rare cause of glomerulonephritis, Alport syndrome must be considered in patients presenting with subnephrotic range proteinuria and microscopic hematuria. Thorough history and physical examination and characteristic findings on kidney biopsy can help in the prompt diagnosis of the disease. Multidisciplinary care and early intervention can improve the quality of life and delay the progression to kidney failure in these patients. In patients who progressively developed to ESRD, kidney transplantation can be offered.

## Data Availability

The data that support the findings of this study are available from the corresponding author upon reasonable request.
